# Hospitals and Medical Schools

**Published:** 1904-11-05

**Authors:** 


### HOSPITAL ADMINISTRATION.

### CONSTRUCTION AND ECONOMICS.

### HOSPITALS AND MEDICAL SCHOOLS.

We have been requested to publish the following correspondence:—

Hay's Wharf and Dock, Southwark,
October 21st, 1904.

The Hon. Stephen Coleridge,
7 Egerton Mansions, South Kensington.

Dear Sir,—I have been spending the autumn in Scotland, and on my return found your letter of the 6th inst. At a meeting of the executive committee on September 30, a discussion took place on the question of the financial relations between the hospitals and the medical schools, and I think it probable some announcement will appear shortly. In the meantime, if you could send me any papers on this special subject I should be obliged.

I remain, your obedient servant,
Hugh C. Smith.

92, Victoria Street, October 27th, 1904.

Dear Sir,—I am obliged to you for your letter. You will no doubt remember that in April last, when I asked you for some information as chairman of King Edward's Hospital Fund, you told me that you declined to be drawn into controversy. I hope therefore now that you invite me to send you information you will not consider that I am disregarding your former wishes if I comply with your present request. I have taken the liberty of expressing my view of the diversions made by hospital managers of the funds committed to their charge, and of the action of King Edward's Hospital Fund Council in countenancing and assisting those diversions, in an article in the forthcoming November number of the *Contemporary Review* entitled "A Great Breach of Trust," an early copy of which I will give myself the pleasure of sending you. I have confined myself in that article to a few figures only taken from the audited accounts of the hospitals concerned, because I thought it essential in a popular paper to avoid too much arithmetic. But had I been addressing myself only to you and your fellow-members of the council of the King's Fund I should have made larger extracts from those accounts. As the audited accounts of all the Metropolitan hospitals are, of course, familiar to you and the other members of the council of King Edward's Fund, you are fully aware that the total amount of money diverted in out-and-out grants from the general funds of four of the largest hospitals, since the fund of which you are chairman undertook its various grave functions, amounts to £26,803 7s.

| | £ | s. | d. |
|---|---|---|---|
| Middlesex ... ... ... ... | 5,312 | 18 | 4 |
| London ... ... ... ... | 15,439 | 14 | 2 |
| Guy's ... ... ... ... ... | 2,864 | 13 | 11 |
| Charing Cross... ... ... ... | 3,186 | 0 | 7 |
| Total ... ... ... ... | £26,803 | 7 | 0 |

It is no doubt well known to you and the council that the audited accounts of the London Hospital . . . . show that the establishment of the King's Fund and the intrusion of its influence and advice on the hospital management synchronises with the appropriations of very large and ever-increasing sums of hospital funds to what are designated as "loans" to the medical school, and credits to an account called "sports ground" in addition to the out-and-out grants of money to the school.

In the accounts to December 31st, 1896, no such loans or credits appear.

In the accounts to December 31st, 1897, no such loans or credits appear.

At the end of 1897 an annual grant of £5,000 and a donation of £3,937 10s. was allocated to the hospital by the King's Fund.

In the accounts to December 31st, 1898, there appears in the accounts:

| | £ | s. | d. |
|---|---|---|---|
| Loan to Medical School... ... | 5,814 | 7 | 0 |

In the accounts to December 31, 1899, there appears in the accounts:

Loans to Medical School.

| | £ | s. | d. |
|---|---|---|---|
| No. 1 ... ... ... ... ... | £8,959 | 6 | 4 |
| No. 2 ... ... ... ... ... | 1,399 | 17 | 8 |
| | £10,359 | 4 | 0 |

In the accounts to December 31, 1900, there appears in the accounts:

Loans to Medical School.

| | £ | s. | d. |
|---|---|---|---|
| No. 1 ... ... ... ... ... | £8,817 | 12 | 2 |
| No. 2 ... ... ... ... ... | 1,760 | 6 | 6 |
| Sports Ground ... ... ... | 3,200 | 0 | 0 |
| | £13.777 | 18 | 8 |

In the accounts to December 31, 1901, there appears in the accounts:

Loans to Medical School.

| | £ | s. | d. |
|---|---|---|---|
| No. 1 ... ... ... ... ... | £8,671 | 5 | 0 |
| No. 2 ... ... ... ... ... | 2,684 | 8 | 11 |
| Sports Ground ... ... ... | 5,371 | 15 | 6 |
| | £16,727 | 9 | 5 |

In the accounts to December 31, 1902, there appears in the accounts:

Loans to Medical School.

| | £ | s. | d. |
|---|---|---|---|
| No. 1 ... ... ... ... ... | £8,587 | 11 | 1 |
| No. 2 ... ... ... ... ... | 2,630 | 4 | 3 |
| Sports Ground... ... ... ... | 6,170 | 18 | 11 |
| | £17,388 | 14 | 3 |

In the accounts to December 31, 1903, there appears in the accounts:

Loans to Medical School.

| | £ | s. | d. |
|---|---|---|---|
| No. 1 ... ... ... ... ... | £8,433 | 4 | 7 |
| No. 2 ... ... ... ... ... | 2 575 | 19 | 7 |
| Sports Ground... ... ... ... | 6,170 | 18 | 11 |
| | £17,180 | 3 | 1 |

Your council must, therefore, be well aware that no one who studies this subject can very well fail to come to the conclusion that it is impossible for the council to escape the responsibility of directly sanctioning, not only out-and-out diversions to the school of hospital funds to the extent of £15,439 14s. 2d. since the establishment of the King's Fund, but also "loans" and credits to the school and sports ground that now have reached the formidable amount of £17,180 3s. 1d.

The Council of the King's Fund have, it would seem, cheerfully taken on themselves the responsibility of sanctioning and approving the transference from the funds of the hospital under one method or another of £32,619 16s. 3d. to purposes other than the tending of the sick poor, and neither the public nor the subscribers to the hospital have ever had any proper accounts furnished them of what has been done with these immense sums of money. If anyone asserts that these loans to the school and credits to the "sports ground" are excellent investments of the hospital's reserve funds on first-class security, it would be interesting to learn from him how and when he imagines the school and its sports ground that needs yearly assistance to the extent of over £2,000 to square its accounts is ever going to repay a sum of £17,180 3s. 1d. Indeed, the published accounts contain nothing to preclude the hypothesis that the hospital's yearly out-and-out grant of money to the school is in part devoted to paying back the interest to itself on the loans it has itself made out of the money belonging to the poor. It is impossible in the compass of a letter to rehearse severally all the instances of misappropriation by other hospitals of funds subscribed exclusively for the poor, each of which must be familiar to, and be approved by, the majority of the Council of the King's Fund.

I believe the time is now not far distant when it will be universally recognised that these misappropriations constitute one of the most serious scandals of our time, jeopardising the continued independent management of our hospitals, and very grievously injuring the sacred interests of the sick poor. The duty laid on me as the person entrusted by a great society with the task of defending helpless animals from torture, has led me incidentally to the discovery that one wrong has inextricably been interwoven with another, and I have found that where animals are vivisected under the patronage of the hospitals, there it is that the poor are robbed of what is theirs by every law of probity and every precept of honour. I deeply regret that so far I have been left to enter my protests alone, and that not one of the great and eminent persons that form the council of the King's Fund has found himself able to say a word in public against the detestable practice of vivisection, and only one has been found to raise his voice in protest against these indefensible financial transactions.

Your obedient servant,

STEPHEN COLERIDGE.

Hugh C. Smith, Esq., Chairman King Edward's Hospital Fund, 81 Cheapside.

.·. The action of the King's Hospital Fund in referring the questions raised in this correspondence to a Special Commission, is referred to in another column of our present issue.—ED. THE HOSPITAL.

---

The attention of King Edward's Hospital Fund for London having for some time past been called to the financial relations of certain of the London hospitals to their medical schools, the question has been referred to a committee which will consist of Lord Welby, Sir Edward Fry, and the Bishop of Stepney. The terms of the reference are as follows:—To consider and report—

(1) Whether any, and if any how much, money given or subscribed for the relief of the sick poor to the 12 London hospitals having medical schools, is contributed, directly or indirectly, by those hospitals, or any of them, for the maintenance of medical education.

(2) Whether any direct or indirect return for such contributions (if any) is received by the hospitals from their medical schools, and, if so, whether such return is equivalent to the amount of the contributions.

(3) Whether, in the event of the committee finding that any hospital contributes to its medical school a sum in excess of the return it receives from the medical school, there are any special considerations advanced in justification of such expenditure, or any general considerations which would apply to all hospitals having medical schools.

It is an instruction to the committee to deal with the subject on the basis of the existing arrangements, and to accept from the hospitals as existing arrangements any such as they may advise the committee will be in operation on January 1st, 1905.

